# Evaluation of the Modal Parameters of a Unidirectional Carbon-Based Composite Structure Using the Influential Factor of Static Loading

**DOI:** 10.3390/ma17133209

**Published:** 2024-07-01

**Authors:** Seunghwan Chung, Chan-Jung Kim

**Affiliations:** 1 Department of Automobile Engineering, Korea Polytechnic Colleges, Seoul 04392, Republic of Korea; shchung@kopo.ac.kr; 2 School of Mechanical Engineering, Pukyoung National University, Busan 48513, Republic of Korea

**Keywords:** unidirectional carbon-based composite structure, static load influential factor, uniaxial static load, modal parameters, carbon fiber orientation

## Abstract

Static loading can significantly alter the dynamics of unidirectional carbon-based composites (UCBCs), with modal parameters varying depending on the orientation of the carbon fibers. In this study, the sensitivity of modal parameters of UCBC structures under uniaxial static loading was investigated. The theoretical static load influential factor was derived from a linearized UCBC model and corresponded to the transformed decoupled response over the mass-normalized static load. Three rectangular UCBC specimens (carbon fiber orientation of 0°, 45°, and 90°) were prepared under fixed–fixed boundary conditions using a jig fixture. Uniaxial static loads between 0 N and 1000 N were applied, and the first three modes of the UCBC specimens were analyzed. An isotropic SUS304 specimen was used as a reference. The linearization assumption about the UCBC structure was preliminarily validated with the Modal Assurance Criterion (MAC). A high influential factor was found for the UCBC specimen when carbon fibers were aligned with the static load direction at the first two resonance frequencies. Therefore, the proposed influential factor is an efficient indicator for determining the sensitivity of the dynamic response of a UCBC structure over a static load case. The variations in the influential factors for the UCBC specimens were more pronounced than for the isotropic specimens.

## 1. Introduction

A carbon-based composite structure was used to maximize the specific structural rigidity relative to the required mass, with the reinforcing carbon-fiber being a critical element within the composite volume. Directional carbon-based composites are pre-impregnated with resin and arranged in thin sheets. These sheets are then stacked together to create a three-dimensional structure. Specialized techniques including autoclave curing, resin transfer molding, and other procedures are used to bind the sheets together [1,2,3,4]. The arrangement of carbon fibers in these pre-impregnated thin sheets has been produced by various weaving methods, including plain, twill, and unidirectional weave [5,6,7]. The choice of the binding matrix is also a critical factor in determining the mechanical properties of carbon-based composite structures in many industries [8,9,10].

The uniaxial carbon-based composite (UCBC) structure represents the basic layout of carbon fibers and is efficient for determining the dynamics of the UCBC structure at different orientations of the carbon fiber. The frequency response function (FRF) is a feasible method to analyze the dynamics of a target structure using modal testing techniques [11,12,13]. This method can be applied to UCBC structures, provided that the nonlinear characteristics of the test specimen can be neglected. UCBC structures may display intricate behavior due to the combination of carbon fibers and the binding matrix in contrast to standard isotropic structures. Hence, the process of linearizing the UCBC structure should be performed using a closely monitored identification approach. Recent studies have investigated the sensitivity of the FRF of UCBC specimens with respect to the spectral loading input pattern, carbon fiber orientation, and operating temperature [14,15]. The modal parameters, including the resonance frequency and mode shape, were calculated from the obtained FRF data. These parameters were derived from the matrices of the physical system by converting the matrix into modal coordinates. Although the modal parameters effectively represent the dynamics of mechanical systems, the linear characteristics of the target system must be preserved during the modeling of the system. The dynamics of the UCBC structure were also identified using the modal parameters of the anisotropic system, and the sensitivity of these parameters to different orientations of the carbon fibers was investigated [16,17,18,19,20,21].

The static loading condition is a common pattern in fatigue-related engineering and this loading condition has been studied for UCBC structures [22,23,24,25]. Previous studies have considered both static and dynamic loads to calculate the fatigue damage or crack propagation in UCBC structures. More recent research has focused on investigating the mechanical properties of carbon-based composite structures using the hysteresis curves between force and displacement [26] and evaluating the strength and buckling characteristics under static loading conditions [27]. When a UCBC structure is subjected to static loading under certain circumstances, such as a background static load or joint coupling with a neighboring system, the dynamics of the original system may change due to the structural deformation caused by the static load. This modification may change the modal parameters of the UCBC structure, thus requiring a refined dynamic identification that takes into account the initial static loading condition.

In this study, a theoretical static load influential factor was proposed based on a linearized UCBC model, and the variations in the modal parameters were formulated as transformed modal displacements with respect to the mass-normalized static load. A high influential factor indicates significant variations in the modal parameters of the target system under a static load at the corresponding resonance frequency. Simple rectangular UCBC specimens were prepared with three orientations of carbon fibers: 0°, 45°, and 90°. In addition, the SUS304 specimen was considered as a reference for the case of isotropic material. The boundary conditions of the specimens were fixed at their ends using clamping jigs. A uniaxial static load was applied to the clamping jig parallel to a 0-degree orientation of the carbon fiber. The load varied from 0 N to 1000 N, increasing by increments of 500 N. The maximum static load of 1000 N was determined based on the equipment’s capacity. The primary objective is to observe the changes in modal parameters with and without the static load; therefore, the physical significance of this maximum value is not considerable. The modal parameters of all specimens were determined by experimental modal testing and used to calculate the static load influential factor for each specimen. The UCBC specimen with a 0-degree orientation of the carbon fiber was the most robust under the assigned static load; however, the influential factor was high for the first two resonance frequencies. Therefore, this specimen was the most susceptible to static loads, necessitating the need for precise dynamic identification to be conducted under static load circumstances. For the three resonance frequencies of interest, the trend of the calculated influential factor changed with increasing the orientation of the carbon fibers; however, the variations in the influential factor were narrow for the isotropic material specimen. Therefore, the proposed static load influential factor is an efficient metric for assessing the variations in modal parameters of UCBC structures.

## 2. Modal Parameters of the UCBC Structure

Although the UCBC structure exhibited a nonlinear response and anisotropic stiffness depending on the orientation of the carbon fibers, the dynamic characteristics of the UCBC structure can be approximately expressed in modal coordinates if the nonlinear factor at the resonance frequency points of interest can be eliminated. Using the linear system model technique, the approximated linear model of a UCBC structure can be verified through several methods, such as the coherence function of the FRF and the modal assurance criterion (MAC) [11,12,13]. Therefore, the feasibility of the linearized system model of the UCBC structure are verified later, and the variations in the dynamics of the UCBC model under the influence of a static load condition are addressed with the approximated linear UCBC model. The linear formulation of the mechanical system can be formulated with three major matrices, mass, damper, and stiffness, and the nth degree of freedom can be denoted by physical variables, as shown in Equation (1) [11,12].

(1) $$M\ddot{X}\left(t\right)+C\dot{X}\left(t\right)+KX\left(t\right)=0$$

Here, $X={\left[\begin{array}{ccc} x_{1}\left(t\right) & \ldots & x_{N}\left(t\right) \end{array}\right]}^{T}$ is the physical column vector of the *n*th degree-of-freedom system; M, C, and K denote the system matrices for mass, damper, and stiffness, respectively; and $\dot{X}\left(t\right)$ and $\ddot{X}\left(t\right)$ are the velocity and acceleration of $X\left(t\right)$. When a static load ($F_{S}$) is applied to the linearized system, the system parameters may differ from the original values, owing to the elastic structural deformation caused by the static loading condition. Except for the mass matrix, the formulation of the linear UCBC structure was changed as follows:

(2) $$M\ddot{X}\left(t\right)+C_{M}\dot{X}\left(t\right)+K_{M}X\left(t\right)=S_{0}$$

Here, $C_{M}$ and $K_{M}$ denote the modified system matrices of the damper and stiffness, respectively. In addition, $S_{0}$ is defined as a diagonal matrix for each physical variable, owing to the nature of the independent static loading input. The previous equations can be simplified to Equation (3) by eliminating the mass matrix in Equations (1) and (2).

(3) $$S_{0}=\left(C_{M}-C\right)\dot{X}\left(t\right)+\left(K_{M}-K\right)X\left(t\right)$$

The physical column vector, $X\left(t\right)$, can be transformed into the modal column vector $R\left(t\right)={\left[\begin{array}{ccc} r_{1}\left(t\right) & \ldots & r_{N}\left(t\right) \end{array}\right]}^{T}$ with a mass-normalized mode shape vector (U), as shown in Equation (4) [11].

(4) $$X\left(t\right)=M^{-1/2}PR\left(t\right)=UR\left(t\right)$$

where P denotes the eigenvector matrix. Equation (3) can then be transformed into modal coordinates as follows:

(5) $$U^{T}S_{0}U=U^{T}\left(C_{M}-C\right)UR\left(t\right)+U^{T}\left(K_{M}-K\right)UR\left(t\right)$$

When a mechanical system is specified for an anisotropic UCBC structure, the system matrices cannot guarantee the symmetric nature of the system matrices as typically found in an isotropic structure. Therefore, the orthogonality of the mass-normalized mode shape vectors of the UCBC structure may not be acceptable in the field. However, the advantage of linearizing the system model is worth considering. The dynamics of the UCBC structure can be identified by obtaining modal parameters, which are valid under the linearized model [11,12]. Therefore, the target UCBC structure is assumed to be a linear system with an allowable margin of system error. If all system parameters, $C_{M}$, C, $K_{M}$, and K, are allowed to be symmetric matrices, the system equation for the UCBC structure can be converted into a decoupled modal coordinate formula, as shown in Equation (6). The left term is simplified as ${M^{-1/2}S}_{0}M^{-1/2}$ because $S_{0}$ is a diagonal matrix and each column vector in U is orthonormal to the other mode vectors [11,12].

(6) $${M^{-1/2}S}_{0}M^{-1/2}=\left[\begin{array}{ccc} 2\Delta \left(\omega_{n,1}\xi_{1}\right) & & zeros\\ & \ddots & \\ zeros & & 2\Delta \left(\omega_{n,N}\xi_{N}\right) \end{array}\right]\dot{R}+\left[\begin{array}{ccc} \Delta {\left(\omega_{n,1}\right)}^{2} & & zeros\\ & \ddots & \\ zeros & & \Delta {\left(\omega_{n,N}\right)}^{2} \end{array}\right]R$$

(7) $$\Delta \left(\omega_{n,i}\xi_{i}\right)=\omega_{n,i}^{M}\xi_{i}^{M}-\omega_{n,i}\xi_{i}$$

(8) $$\Delta \left({\omega_{n,i}}^{2}\right)={\left(\omega_{n,i}^{M}\right)}^{2}-{\left(\omega_{n,i}\right)}^{2}$$

Here, both $\omega_{n,i}$, $\omega_{n,i}^{M}$, and $\xi_{i}$, $\xi_{i}^{M}$ are defined as the ith resonance frequency and modal damping ratio of the UCBC structure in Equations (1) and (2), respectively. Considering the decoupled condition in the modal coordinates, the ith mass-normalized static matrix element (${\hat{s}}_{i}=s_{i}/m_{i}$) can be simplified as follows:

(9) $${\hat{s}}_{i}=2\Delta \left(\omega_{n,i}\xi_{i}\right)\dot{r_{i}}\left(t\right)+\Delta \left({\omega_{n,i}}^{2}\right)r_{i}\left(t\right)$$

where $m_{i}$ is the ith element in the mass matrix M. The frequency of interest is the ith resonance frequency $\omega_{n,i}$ and the response at the resonance frequency point can be expressed as complex formula in Equation (10). This complex value is derived without initial displacement or velocity and j represents an imaginary unit.

(10) $$\frac{r_{i}}{{\hat{s}}_{i}}=\frac{1}{\Delta \left({\omega_{n,i}}^{2}\right)+2\Delta \left(\omega_{n,i}\xi_{i}\right)\omega_{n,i}j}$$

The expression in Equation (10) represents the modal column vector, $r_{i}$, with respect to the mass-normalized static load. The magnitude of this complex value is proportional to the sensitivity of dynamic response to a static load at the resonance frequency of interest at $\omega_{n,i}$. Therefore, the absolute value is a scalar value at the ith resonance frequency of interest of the UCBC structure so that it is defined as the influential factor of the static load ($I_{f,i}$) as follows:

(11) $$I_{f,i}={\left[{\left(2\Delta \left(\omega_{n,i}\xi_{i}\right)\omega_{n,i}\right)}^{2}+\Delta {\left({\omega_{n,i}}^{2}\right)}^{2}\right]}^{\frac{-1}{2}}$$

## 3. Identification of the Modal Parameters of the UCBC Structure

**Figure 1:**
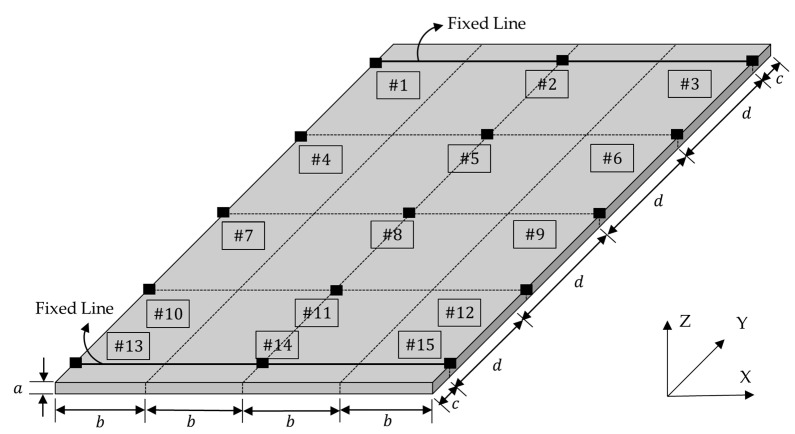
Configuration of the simple rectangular specimen and boundary conditions: a: 3 mm, b: 20 mm, c: 10 mm, d: 32.5 mm.

**Figure 2:**
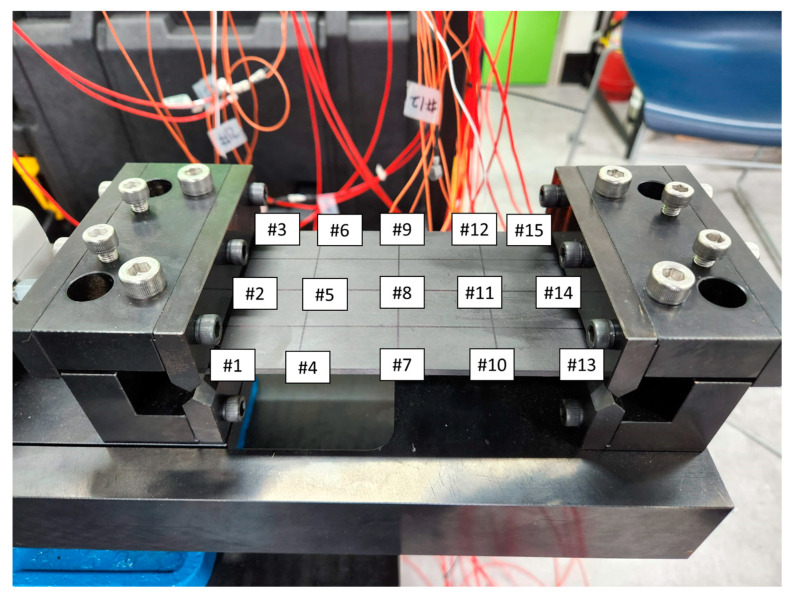
UCBC specimen with fixed–fixed boundary condition.

**Figure 3:**
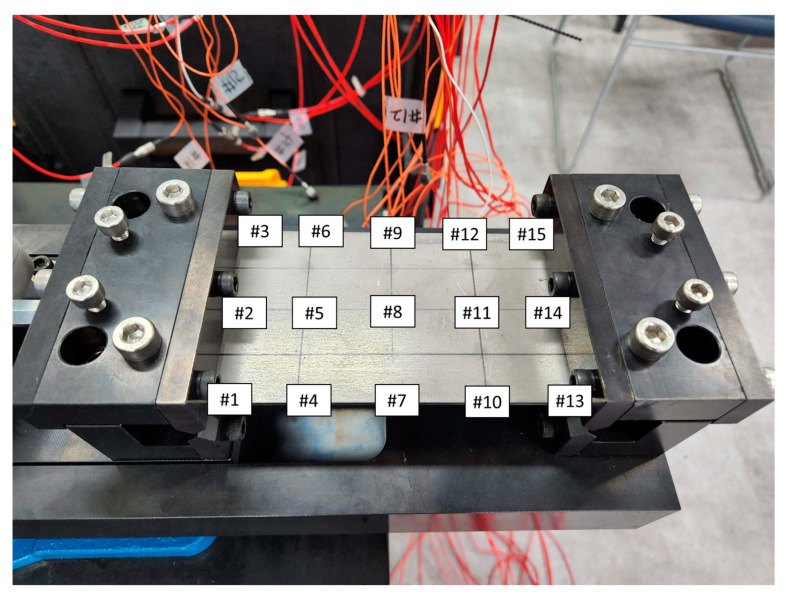
Isotropic specimen (SUS 304) with fixed–fixed boundary condition.

To obtain the modal parameters of UCBC structure under a static load, an experimental modal test was conducted for all specimens. Notably, there is no standard configuration for the test specimens or boundary conditions [12]. A specimen of the UCBC structure was prepared, which had a simple rectangular shape (L × W × T: 150 mm × 80 mm × 3 mm), as shown in Figure 1. Isotropic material (SUS304) was prepared as a reference. To apply a static load to the specimens, a mechanical fixture was designed to clamp both ends of the specimen, and fixed conditions were set for the line constraints (fixed–fixed boundary condition). The accelerometers were placed at equal intervals (#1~#15) on the surface of the specimens. The experimental setup for the simple specimens is shown in Figure 2 and Figure 3.

**Figure 4:**
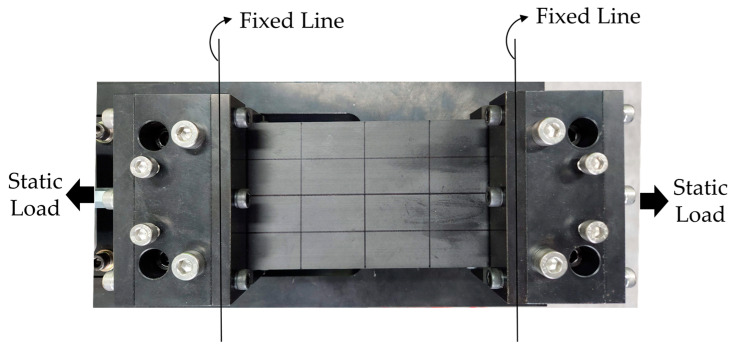
Test setup of the UCBC specimen with a uniaxial static load.

The static loading case was assigned in the uniaxial direction orthogonal to the fixed line so that a stable static load of up to 1000 N could be assigned to the fixed–fixed specimen. The experimental setup is illustrated in Figure 4.

**Figure 5:**
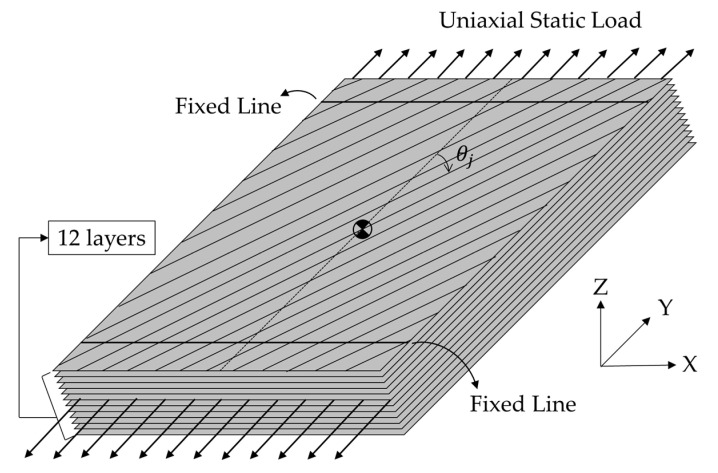
Simple rectangular UCBC specimen with 12-layer UD prepreg with the carbon fiber orientation $\theta_{j}$.

UCBC specimens were prepared for three different orientations of carbon fibers to account for the nature of anisotropic mechanical dynamics. The thin unidirectional pre-implemented (UD prepreg) USN 250A (SK Chemical, Seongnam, the Republic of Korea) was stacked in an autoclave curing process (125 °C maximum temperature) to form a large-scale 12-layer UCBC plate and cut into rectangular simple specimens with three orientations: $\theta_{1}=0{}^{\circ}$, $\theta_{2}=45{}^{\circ}$, ${and\;\theta }_{5}=90{}^{\circ}$. The reference line for the orientation of the carbon fibers was parallel to the direction of the uniaxial static load, and the actual uniaxial static load was valid at the fixed line of the specimens. As shown in Figure 5.

To measure the response acceleration of the UCBC specimen, uniaxial accelerometers (+Z) were attached to the UCBC specimen in Figure 1. Considering the light weight of the specimen, all attached accelerometers (model: 3225F2, Dytran, Chatsworth, CA, USA) had a very low weight (each accelerometer: 1 g) with respect to the UCBC specimen under test (56.5 g) so that the effect of mass loading could be eliminated as much as possible.

The modal parameters, that is, both the resonance frequency and the mode shape vector, can be determined using the experimental mode-testing technique. Under the fixed–fixed boundary condition, the frequency response functions were measured using an impact hammer (model: 5800SL, Dytran, Chatsworth, CA, USA), and the acceleration responses were determined at 15 sensor locations (#1–#15 in Figure 2 and Figure 3). The selected impact hammer has a low mass of 9.8 g and very high stiffness, enabling a spectral input frequency of over 10,000 Hz with a single-stroke impact force assignment. The frequency response functions (FRFs) were obtained through an averaging process of at least 10 impact hammer tests, and the level of impact force was adjusted to consider the response accelerations of the tested specimens. The measurement of FRFs and the identification of modal parameters were performed using Test Lab (Siemens, Munich, Germany), with the frequency range of interest set from 10 to 6400 Hz.

**Figure 6:**
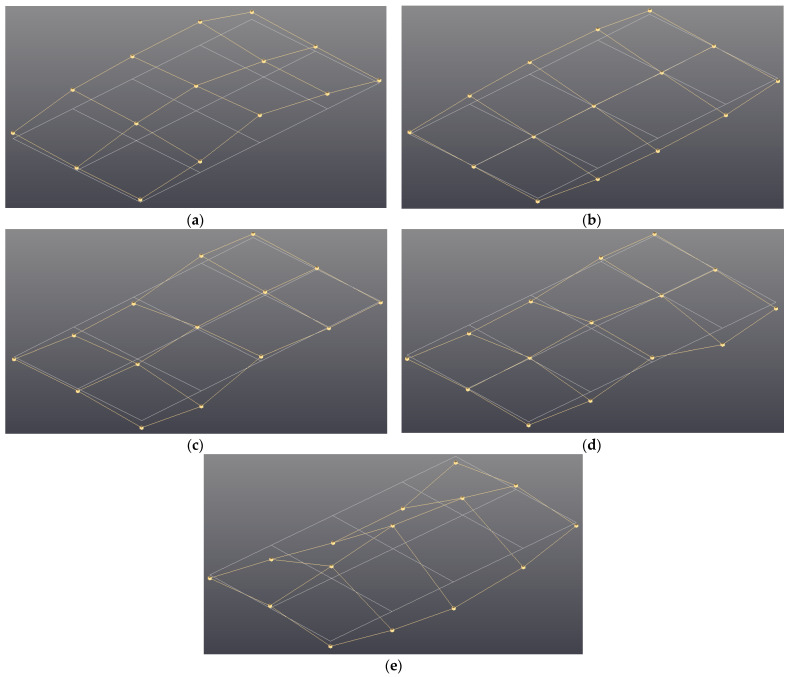
Model shape of SUS304 specimen. Deformed mode shape (yellow color) is superimposed on the original undeformed geometry (white color): (**a**) first mode (first bending); (**b**) second mode (first torsion); (**c**) third mode (second bending); (**d**) fourth mode (second torsion); (**e**) fifth mode (third bending).

The modal parameters of the specimens consisted of four specimens: isotropic SUS304 and three UCBC specimens with carbon fiber orientations of $0^{{}^{\circ}}$, $45^{{}^{\circ}}$, $90^{{}^{\circ}}$, respectively. The specimens were subjected to three static loads of 0, 500, and 1000 N under the fixed–fixed boundary condition. With the help of the PolyMAX algorithm in the Test.Lab software (ver.15), a reliable modal parameter can be guaranteed. The results are summarized in Table 1 and Table 2. Here, B(i) and T(i) denote the ith bending and torsion modes of the specimens, respectively. The reliable linearity of the FRFs was achieved with a high coherence function value (greater than 0.95) at the resonance frequencies of interest. The Modal Assurance Criterion (MAC) value of two identified mode shape vectors is another important indicator for validating the modal parameters of all specimens. An MAC value less than 0.2 is generally considered acceptable for verifying the orthogonality of two independent mode shape vectors. The identified modal parameters are deemed reliable due to the low MAC values in off-diagonal elements, which are less than 0.12, as summarized in Table 3. Therefore, the linearization assumption about the UCBC structure in Equation (1) is acceptable, owing to the reliable validation of modal parameters, and the proposed static load influential factor can also be supported by the experimental results. As the mode order increased, the mode shapes of the specimens became more complicated, making it difficult to identify them with the limited measurement points (#1–#15). Therefore, the order numbers of the higher-order modes cannot be given in Table 1 and Table 2. The ith modal parameters, resonance frequency and modal damping ratio, without static load (0 N) are equivalent to $\omega_{n,i}$ and $\xi_{i}$, respectively. The modal parameters with static loads of 500 N and 1000 N correspond to the modified resonance frequency ($\omega_{n,i}^{M}$) and the modified modal damping ratio ($\xi_{i}^{M}$), respectively. The five selected mode shapes of the isotropic (SUS403) specimens are shown in Figure 6.

## 4. Analysis of the Static Load Influential Factor of the UCBC Structure

The static load influential factor in Equation (11) is derived from the variation in the modal parameters over the static load such that the calculated influential factor is proportional to the change in the system matrices (stiffness and damping) according to the assigned static load condition. In addition, the ith influential factor is the formula for the ith transformed response with respect to the mass-normalized static load at the resonance frequency of interest. Therefore, a high value of the static load influential factor indicates variations in the modal parameters of the target system that lead to variations in the response displacement at the frequency of interest.

**Figure 7:**
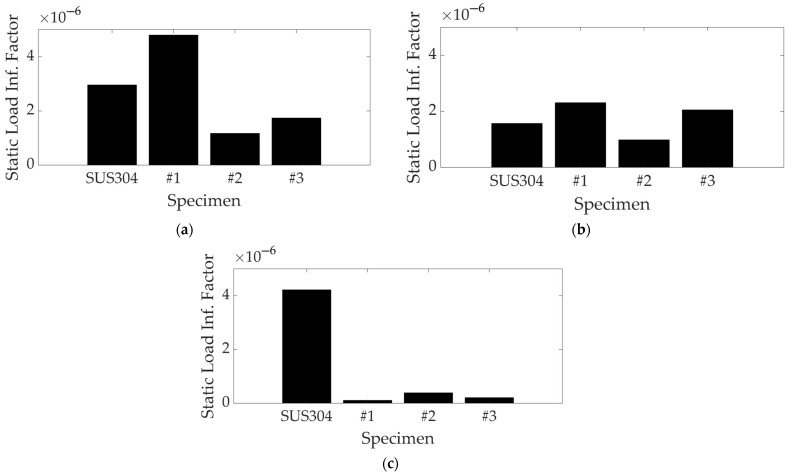
Comparison of static load influential load factors of specimens: (**a**) first mode (first bending); (**b**) second mode (first torsion); (**c**) third mode (second bending).

The static load influence factor in Equation (11) can be calculated using the modal parameters listed in Table 1 and Table 2: the original modal parameters ($\omega_{n,i}$, $\xi_{i}$) were chosen for the static load case of 0 N and the modified modal parameters ($\omega_{n,i}^{M}$, $\xi_{i}^{M}$) with static load case were set for 1000 N, which corresponded to the maximum static load case. In the case of 500 N, the variation in the modal parameters was generally lower than those of 1000 N, so this case was not considered for the calculation of the influential factor. Variations in the two modal parameters were calculated using Equations (7) and (8) and the influential factors for all specimens were finally derived from Equation (11). The calculation results for the static load influential factors of the specimens are summarized in Table 4, and a comparison of the influential factors in each mode is illustrated in Figure 7. Here, the interesting modes of the specimens were limited to the third mode because the model tracking of the UCBC specimens in a higher mode was difficult, owing to the complicated behavior of the mode shape.

The value of the static load influential factor is proportional to the modal column vector with respect to the mass-normalized static load. Consequently, the value of the influential factor will increase if the sensitivity of the dynamic response of the target specimen is high under the static load assignment. Among the UCBC specimens, the static load influential factor of the UCBC #1 specimen was greatest for both the first (first bending) and second (first torsion) modes but was minimal in the third mode (second bending). The influential factor for UCBC #2 was minimal in the first two modes, but its rank increased in the third mode. Specimen UCBC #3 ranked second in all three modes. These results indicate that the influential factor of the UCBC specimens were sensitive to the orientation of the carbon fibers. In contrast, the influential factor of the isotropic SUS304 specimen was highly ranked in all three modes with minimal variation.

Specimen UCBC #1 was the most robust for the assigned static load case, owing to the same orientation of the carbon fibers. However, its initial dynamic behavior was highly susceptible to the assigned static load at the first two resonance frequencies. These trends changed as the orientation of the carbon fibers increased, and the variation in the modal parameters of the UCBC specimens was the main reason for the changes in the influential factors. The variations in the static load influential factor may be related to the mode shape or nodal line of each specimen, which could be correlated with the mode tracking of the UCBC structure, as mentioned in a previous study. For the structure made of isotropic material, the static load influential factor was significant in all three modes, with minor deviations compared to the UCBC specimens. Conversely, the influential factors of the UCBC specimens decreased significantly in all three modes. Thus, it may be inferred that the isotropic material structure exhibited significant dynamic fluctuations when subjected to static loads. However, a critical case was observed in UCBC structures when the orientation of the carbon fiber coincided with the direction of the static load. It is noteworthy that the proposed influential factor for UCBC structures is very sensitive to the orientation of the carbon fibers. This suggests that a comprehensive examination of the changed system dynamics of UCBC structures under static loading circumstances is necessary.

These results for the influential factor under static loading apply to the measured modal parameters obtained by the experimental modal tests. If the test specimen exhibits highly nonlinear dynamic behavior under operating conditions, the general modal test procedure may distort the actual physical properties of the UCBC structure. Considering the difficulties in the mode-tracking of UCBC structures, the analysis was limited to the first three modes (two bending and one torsion) [19,20]. In addition, a maximum static load of 1000 N was applied to the ends of the specimen under fixed–fixed boundary conditions. Consequently, the nature of the discussion may change depending on different boundary conditions and static load scenarios.

## 5. Conclusions

A theoretical static load influential factor was proposed to evaluate the variation in the modal parameters of the UCBC structure when subjected to static loading. The influential factor was derived under the assumption of a linear model of the UCBC structure, which was validated using the coherence function of FRFs and MAC values. It was assumed that the original system parameters, including the stiffness and damping matrices, changed due to the assigned static load condition. The ith static load influential factor was formulated as the transformed response with respect to the mass-normalized static load at the ith resonance frequency. The dynamics of the UCBC structure were assumed to be linear, and modal tests were performed on three UCBC specimens (with carbon fiber orientations of 0°, 45°, and 90°) and one SUS304 specimen. From the determined modal parameters, the proposed static load influential factor was calculated and analyzed for the first three modes (two bending and one torsion). The boundary conditions were fixed–fixed at the ends of the specimens, and a static load was applied uniaxially in a direction orthogonal to the constraint line of the clamping jig.

Under the uniaxial static load condition, UCBC #1 was the most robust and exhibited the highest static load influential factor in the first two modes. This indicated that the dynamic response of the UCBC structure to a static load was most sensitive in this resonance range. The proposed static load influential factors are proportional to the sensitivity of the transformed decoupled response to the mass-normalized static load when subjected to a static load. As a result, they are well suited for predicting the dynamic sensitivity of the UCBC structure under an assigned static loading case. Other UCBC specimens showed different values of the influential factors, and there was no discernible pattern observed as the carbon fiber orientation increased. The influential factor may be closely related to the mode shape (or nodal line) of each specimen, which necessitates the inclusion of a comprehensive mode-tracking technique for UCBC specimens in future work.

## Figures and Tables

**Table 1:** Modal parameters of the SUS304 specimen.

Static Load	0 N		500 N		1000 N	
Modal Parameter	Resonance Freq. (Hz)/Mode	Damping Ratio (%)	Resonance Freq. (Hz)/Mode	Damping Ratio (%)	Resonance Freq. (Hz)/Mode	Damping Ratio (%)
SUS304	431.3 9/B(1)	2.7	443.2/B(1)	1.8	441.1/B(1)	2.7
	932.1/T(1)	1.5	934.4/T(1)	0.7	937.8/T(1)	0.8
	1468.4/B(2)	1.4	1458.8/B(2)	1.1	1467.0/B(2)	1.3
	2149.3/T(2)	1.9	2116.3/T(2)	1.9	2133.2/T(2)	1.4
	2831.5/B(3)	1.9	2841.7/B(3)	1.7	2833.6/B(3)	2.7
	3111.4/B	1.4	3095.9/B	2.1	3132.1/B	2.3

**Table 2:** Modal parameters of the three UCBC specimens.

Static Load	0 N		500 N		1000 N	
Modal Parameter	Resonance Freq. (Hz)/Mode	Damping Ratio (%)	Resonance Freq. (Hz)/Mode	Damping Ratio (%)	Resonance Freq. (Hz)/Mode	Damping Ratio (%)
UCBC #1 $\left(\theta_{1}=0{}^{\circ}\right)$	756.5/B(1)	1.9	763.1/B(1)	3.1	759.6/B(1)	2.1
	871.3/T(1)	2.9	876.0/T(1)	3.1	874.6/T(1)	3.5
	1386.7/B(3)	3.8	1397.1/B(3)	4.0	1399.2/B(3)	4.0
	2320.9/B(2)	1.4	2295.2/B(2)	2.9	2313.0/B(2)	3.5
	2523.4/B	4.3	2519.5/B	5.4	2546.3/B	2.2
UCBC #2 $\left(\theta_{1}=45{}^{\circ}\right)$	393.7/B(1)	3.2	405.9/B(1)	2.6	420.0/B(1)	2.2
	699.1/T(1)	2.3	706.7/T(1)	2.1	716.8/T(1)	2.8
	1103.4/B(2)	2.8	1120.4/B(2)	2.8	1132.3/B(2)	2.6
	1283.4/T(3)	4.2	1288.3/T(3)	3.9	1295.2/T(3)	4.0
	1923.1/B	4.1	1903.5/B	4.6	1896.3/B	4.3
	2153.7/B	2.1	2160.1/B	4.2	2155.8/B	3.4
	2532.7/B	3.8	2413.5/B	3.4	2404.4/B	2.9
UCBC #3 $\left(\theta_{1}=90{}^{\circ}\right)$	324.3/B(1)	3.7	325.7/B(1)	2.2	344.4/B(1)	1.0
	499.2/T(1)	3.6	479.3/T(1)	2.7	487.5/T(1)	2.8
	1534.5/B(2)	4.7	1533.0/B(2)	3.4	1559.2/B(2)	2.7
	2127.6/T(2)	5.4	2293.2/T(2)	3.3	2271.7/T(2)	2.2
	3451.4/B(3)	3.2	3424.4/B(3)	3.0	3411.7/B(3)	3.1
	4168.3/B	3.6	4155.5/B	2.9	4076.9/B	3.5

**Table 3:** MAC values of specimens (unit: %).

SUS304				UCBC #1			
	B(1)	T(1)	B(2)		B(1)	T(1)	B(2)
B(1)	1.00	0.05	0.08	B(1)	1.00	0.05	0.06
T(1)	0.05	1.00	0.10	T(1)	0.05	1.00	0.08
B(2)	0.08	0.10	1.00	B(2)	0.06	0.08	1.00
**UCBC #2**				**UCBC #3**			
	**B(1)**	**T(1)**	**B(2)**		**B(1)**	**T(1)**	**B(2)**
B(1)	1.00	0.06	0.03	B(1)	1.00	0.04	0.10
T(1)	0.06	1.00	0.12	T(1)	0.04	1.00	0.12
B(2)	0.03	0.12	1.00	B(2)	0.10	0.12	1.00

**Table 4:** Static load influential factors of test specimens.

Specimen	Mode	Static Load Influential Factor ($\times 10^{-4}$)
UCBC #1 $\left(\theta_{1}=0{}^{\circ}\right)$	#1	1.90
	#2	0.91
	#3	0.04
UCBC #2 $\left(\theta_{1}=45{}^{\circ}\right)$	#1	0.46
	#2	0.39
	#3	0.15
UCBC #3 $\left(\theta_{1}=90{}^{\circ}\right)$	#1	0.69
	#2	0.81
	#3	0.08
SUS304	#1	1.17
	#2	0.62
	#3	1.67

## Data Availability

The original contributions presented in the study are included in the article, further inquiries can be directed to the corresponding author.
